# Oral Administration of *P*. *gingivalis* Induces Dysbiosis of Gut Microbiota and Impaired Barrier Function Leading to Dissemination of Enterobacteria to the Liver

**DOI:** 10.1371/journal.pone.0134234

**Published:** 2015-07-28

**Authors:** Mayuka Nakajima, Kei Arimatsu, Tamotsu Kato, Yumi Matsuda, Takayoshi Minagawa, Naoki Takahashi, Hiroshi Ohno, Kazuhisa Yamazaki

**Affiliations:** 1 Laboratory of Periodontology and Immunology, Division of Oral Science for Health Promotion, Niigata University Graduate School of Medical and Dental Sciences, Niigata, Japan; 2 Division of Periodontology, Department of Oral Biological Science, Niigata University Graduate School of Medical and Dental Sciences, Niigata, Japan; 3 Laboratory for Intestinal Ecosystem, RIKEN Center for Integrative Medical Sciences (IMS), Yokohama, Japan; 4 CREST, Japan Science and Technology Agency, Kawaguchi, Japan; GI Lab, UNITED STATES

## Abstract

Although periodontitis has been implicated as a risk factor for various systemic diseases, the precise mechanisms by which periodontitis induces systemic disease remain to be elucidated. We have previously revealed that repeated oral administration of *Porphyromonas gingivalis* elicits endotoxemia via changes in the gut microbiota of the ileum, and thereby induces systemic inflammation and insulin resistance. However, it is not clear to what extent a single administration of *P*. *gingivalis* could affect gut microbiota composition, gut barrier function, and subsequent influx of gut microbiota into the liver. Therefore, in the present study, C57BL/6 mice were orally administered *P*. *gingivalis* (strain W83) once and compared to sham-inoculated mice. The phylogenetic structure and diversity of microbial communities in the gut and liver were analyzed by pyrosequencing the 16S ribosomal RNA genes. Serum endotoxin activity was determined by a Limulus amebocyte lysate test. Gene expression in the intestine and expression of 16S rRNA genes in the blood and liver were examined by quantitative polymerase chain reaction. Administration of *P*. *gingivalis* significantly altered gut microbiota, with an increased proportion of phylum Bacteroidetes, a decreased proportion of phylum Firmicutes, and increased serum endotoxin levels. In the intestinal tissues, gene expression of tjp-1 and occludin, which are involved in intestinal permeability, were downregulated. Higher amounts of bacterial DNA were detected in the liver of infected mice. Importantly, changes in gut microbiota preceded systemic inflammatory changes. These results further support the idea that disturbance of the gut microbiota composition by orally derived periodontopathic bacteria may be a causal mechanism linking periodontitis and systemic disease.

## Introduction

Periodontal disease is a group of chronic inflammatory diseases resulting from dysbiosis of oral microbiota. Epidemiological studies indicate its association with increased risk of various diseases such as type 2 diabetes mellitus[1, 2], atherosclerotic vascular diseases [3, 4], and rheumatoid arthritis [5]. Although it is possible that common disease susceptibilities and risk factors could explain the association between these diseases, endotoxemia, proinflammatory cytokines, and molecular mimicry have been considered causal mechanisms [6]. However, compelling evidence is still lacking.

Interestingly, the diseases reported to be affected by periodontal disease are often described in association with dysbiosis of the gut microbiota [7, 8]. Under physiological conditions, bacteria in the intestine are commensal and mediate food digestion, strengthen the immune system, and prevent pathogens from invading tissues and organs. However, once dysbiosis occurs and detrimental bacteria become predominant, noxious agents such as bacterial toxins and metabolites can damage the gut epithelial wall. These are then absorbed into the systemic circulation through the disrupted epithelium, resulting in impairment of various tissues and organs such as the liver, heart, kidney, pancreas, and blood vessels [9]. Despite this, there is a question as to whether changes in gut microbiota alter physiological conditions such as the host immune system or if immunological and inflammatory changes induce the alteration of the gut microbiota composition. Examining this connection, Vijay-Kumar *et al*. demonstrated that Toll-like receptor 5 knockout (Tlr5^-/-^) mice develop metabolic syndrome, and that the transfer of Tlr5^-/-^ gut microbiota into wild-type mice confers many aspects of the Tlr5^-/-^ phenotype to the recipient mice [10]. These results suggest that physiologic conditions and the composition of gut microbiota could affect one another.

We have recently demonstrated that repeated oral administration of *Porphyromonas gingivalis* resulted in alteration of the gut bacterial composition, which coincided with inflammatory changes in the adipose tissue and liver. Since the gut microbial change is associated with decreased gut barrier function, as evidenced by reduced expression of the tight junction protein gene and elevated endotoxin levels in the systemic circulation, the inflammation in the adipose tissue and liver was expected to be caused by an influx of bacteria and/or bacterial products into these tissues. In addition, in this mouse model, the administered *P*. *gingivalis* was not found in the blood, and only little inflammation was obvious in gingival tissue, suggesting that conventional causal mechanisms for the link between periodontitis and systemic diseases may not be applicable at least in this model [11]. In addition, it is reported that in patients with liver cirrhosis, major changes in the gut microbiota are attributable to massive invasions of the gut by oral bacterial species [12]. Moreover, a higher load of diverse bacteria, including enterobacteria, has been detected in biopsies from patients with various vascular diseases comorbid with chronic periodontitis (CP), compared with those without CP [13].

However, it is not clear to what extent a single administration of *P*. *gingivalis* could affect gut microbiota composition, gut barrier function, and subsequent influx of gut microbiota into the liver.

We show in the present study that even only a single administration of *P*. *gingivalis* has a significant impact on the gut microbiota composition and gut barrier function, resulting in the dissemination of enterobacteria to the liver and endotoxemia. Our results provide further insight into the role of periodontopathic bacteria-mediated gut microbiota modulation in systemic disease.

## Materials and Methods

### Ethics Statement

This study was approved by the Institutional Animal Care and Use Committee at Niigata University (permit number 61–4). All experiments were performed in accordance with the Regulations and Guidelines on Scientific and Ethical Care and Use of Laboratory Animals of the Science Council of Japan, enforced on June 1, 2006.

### Mice

Six-week-old male C57BL/6 mice were obtained from Japan SLC, Inc. (Shizuoka, Japan). The mice were acclimatized under specific pathogen-free conditions and fed regular chow and sterile water until the commencement of infection at 7 weeks of age.

### Bacterial cultures

*P*. *gingivalis* (strain W83) was cultured in modified Gifu anaerobic medium (GAM) broth (Nissui, Tokyo, Japan) in an anaerobic jar (Becton Dickinson Microbiology System, Cockeysville, MD) in the presence of an AnaeroPack (Mitsubishi Gas Chemical Co. Inc., Tokyo, Japan) for 48 hrs at 37°C. Bacterial suspensions were prepared in phosphate-buffered saline (PBS) without Mg^2+^/Ca^2+^ using established growth curves and spectrophotometric analysis. The number of colony-forming units (CFUs) was standardized by measuring optical density (600 nm).

### Oral administration

A total of 10^9^ CFUs of live *P*. *gingivalis* suspended in 100 μL PBS with 2% carboxymethyl cellulose (Sigma-Aldrich, St. Louis, MO) was given to the oral cavity of each mouse through a feeding needle. The number of bacteria to administer was determined by considering the body weight and the number of bacteria in the saliva of periodontitis patients. The control group was sham-administered 100 μL PBS with 2% carboxymethyl cellulose without *P*. *gingivalis*. After administration, all mice were allowed to eat and drink *ad libitum*. Mice were euthanized with CO_2_ at 3, 24, or 48 hrs after *P*. *gingivalis* administration, and their tissues were collected.

### DNA extraction from samples

DNA was extracted from feces as described previously [14]. In brief, feces were collected 6, 24, and 48 hrs after the administration of *P*. *gingivalis*. The freeze-dried feces for 16S rRNA gene sequencing were suspended with buffer [10% sodium dodecyl sulphate / 10 mM Tris-HCl, 1mM EDTA, pH 8.0]. Feces in mixture buffer were disrupted with 0.1 mm zirconia/silica beads (BioSpec Products, Inc., Bartlesville, OK) by shaking (1500 rpm, for 10 min) using ShakeMaster (Hirata Co., Tokyo, Japan). After centrifugation, the bacterial DNA was purified using phenol /chloroform /isoamyl alcohol (25: 24: 1) solution. The DNA was precipitated by adding ethanol and sodium acetate. RNase treatment and polyethylene glycol precipitation was performed.

Whole blood and liver specimens were collected at 3, 24, and 48 hrs after the administration of *P*. *gingivalis*. DNA from these samples was extracted using a QIAampDNA Blood Mini Kit (Qiagen, Hilden, Germany).

### Detection of *P*. *gingivalis* in blood and feces

Quantitative real-time PCR was performed on a LightCycler 96 System (Roche, Basel, Switzerland) using FastStart Essential DNA Green Master (Roche). Universal 16S rRNA was amplified using the forward primer 5′-ACTCCTACGGGAGGCAGCAGT-3′ and the reverse primer 5′-ATTACCGCGGCTGCTGGC-3′. *P*. *gingivalis* 16S rRNA was amplified using the forward primer 5′-AGGCAGCTTGCCATACTGCG-3′and the reverse primer 5′-ACTGTTAGCAACTACCGATGT-3′.

### Gut and liver microbiota analysis by 16S rRNA sequencing

The V4 variable region (515F-806R) of the respective samples was sequenced on Illumina Miseq, following the method of Kozich *et al*. [15]. Each reaction mixture contained 15 pmol of each primer, 0.2 mM deoxyribonucleoside triphosphates, 5 μl of 10×Ex Taq HS buffer, 1.25 U Ex Taq HS polymerase (Takara Bio, Inc., Shiga, Japan), 50 ng extracted DNA and sterilized water to achieve a final volume of 50 μl. PCR program was set as follows: 95°C 2 min and 25 cycles of 95°C 20 s, 55°C 15 s, 72°C 5 min followed by 72°C for 10 min.

The PCR product was purified by AMPure XP (Beckman Coulter, Inc., Brea, CA), and quantified using Quant-iT PicoGreen ds DNA Assay Kit (Lifi Technologies Japan, Ltd, Tokyo, Japan). Mixed samples were prepared by pooling approximately equal amounts of PCR amplicons from each sample. The pooled library was analyzed with the Agilent High Sensitivity DNA Kit on an Agilent 2100 Bioanalyzer. Realtime PCR for quantification was performed on pooled library using the KAPA Library Quantification Kit for Illumina following manufacture provide protocols.

Based on the quantification, the sample library was denatured and diluted. Sample library with 20% denatured PhiX spike-in was sequenced by Miseq using 500 cycles kit. We obtained 2×250 bp paired-end reads.

Taxonomic assignments and estimating relative abundance of sequencing data were performed using the analysis pipeline of the QIIME software package [16]. An operational taxonomic unit (OTU) was defined at 97% similarity. OTUs indicating relative abundance of under 0.1% were filtered to remove noise. The OTU was assigned taxonomy based on comparison to the Silva database using UCLUST [17, 18]. Rarefaction plots with OTU-based measure (Chao1 metric) were employed to evaluate the amount of diversity contained within communities. Chao1 richness estimation was calculated using the Qiime pipeline. The OTU table was rarefied to the sample with the least reads for each sample. Alpha diversity measures were calculated 10 times at each depth. The rarefaction depth was set in total 10 steps from 10 reads. Principal coordinate analysis (PCoA) using weighted or unweighted UniFrac distance based on OUT distribution across samples was performed to provide an overview of gut microbial dynamics in response to *P*. *gingivalis* administration [19].

### Endotoxin assay

Endotoxin levels were determined in sera collected 48 hrs after the administration of *P*. *gingivalis* using a Limulus amebocyte lysate test (QCL-1000TM, BioWhittaker, Walkersville, MD) according to the manufacturer’s instructions. Serum samples were diluted 1-to-4 for the assay. Optical densities were measured using an ELISA plate reader (Model 680, Bio-Rad Laboratories, Hercules, CA) at 405 nm.

### Analysis of gene expression in the intestine

Total RNA from the small and large intestines collected 24 hrs and 48 hrs after the administration of *P*. *gingivalis* was extracted using TRI Reagent (Molecular Research Center, Inc., Cincinnati, OH). cDNA was synthesized with Transcriptor Universal cDNA Master (Roche Molecular Systems, Inc., Branchburg, NJ). Primers and probes specific for real-time PCR were purchased from Life Technologies Corporation. Reactions were carried out in a final volume of 25 μL in a LightCycler 96 System (Roche) using TaqMan Gene Expression Assays (Life Technologies Corporation) containing 900 nM each of the forward and reverse primers and a 250 nM probe. The reactions consisted of a 10-min incubation at 95°C, followed by 45 cycles of a 2-step amplification procedure consisting of annealing/extension at 60°C for 1 min and denaturation for 15 s at 95°C. LightCycler 96 software (Roche) was used to analyze the standards and carry out the quantification. The relative quantity of each mRNA was normalized to the relative quantity of glyceraldehyde-3-phosphate dehydrogenase (GAPDH) mRNA.

### Statistical analysis

Nonparametric data were evaluated by the Mann-Whitney U-test for two-group comparisons using GraphPad Prism (GraphPad Software, Inc., La Jolla, CA). Analysis of similarity (ANOSIM) was performed to test the differences in the bacterial community compositions across different groups (e.g., *P*. *gingivalis*-administered and sham-administered samples) using the vegan package in R (http://cran.at.r-project.org/). It calculates R as a statistic value that describes the level of similarity between each pair in the ANOSIM. Values close to unity indicate completely different communities in two groups, while a zero value indicates complete overlap or similarity (null hypothesis). A probability value of p < 0.05 was considered statistically significant.

## Results

### *P*. *gingivalis* administration alters the gut microbiota

**Fig 1:**
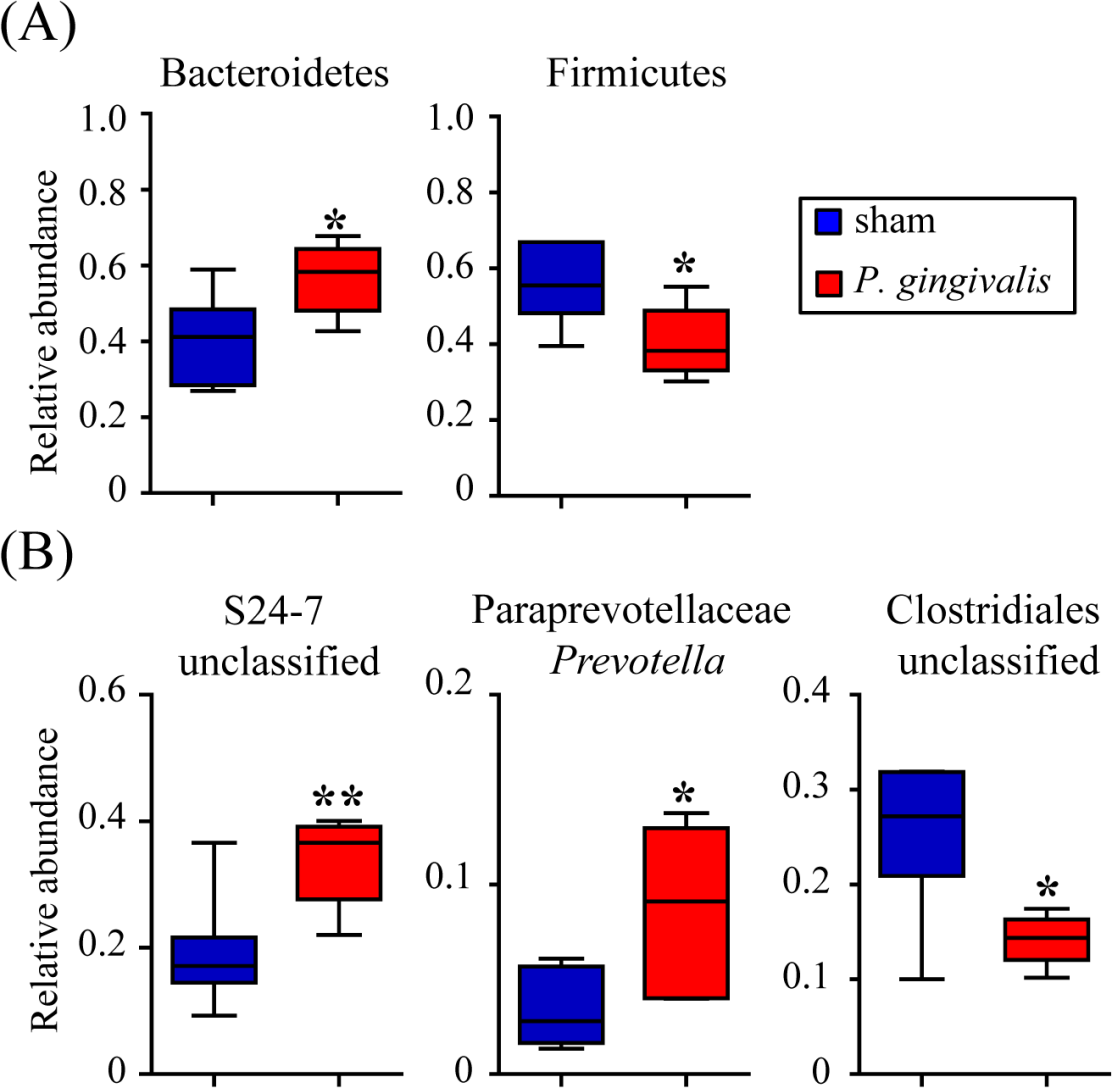
Comparison of the gut microbiota between *P*. *gingivalis*-administered and sham-administered mice by 16S rRNA sequencing analysis (N = 6 in the experimental group, N = 7 in the control group). (A) Relative abundances of each bacterial group at the phylum level at 24 hrs and 48 hrs after *P*. *gingivalis* administration are indicated by box plots. (*p < 0.05, **p < 0.01, Mann-Whitney U-test). (B) Relative abundance of each bacterial group at the genus level were significantly increased or decreased are shown. Each assigned taxonomy obtained from 16S rRNA deep sequencing was compared to Silva database using UCLUST. (*p < 0.05, **p < 0.01, Mann-Whitney U-test).

At the phylum level, no differences in the proportions of Firmicutes or Bacteroidetes were observed in samples obtained at 0 and 3–6 hrs between *P*. *gingivalis*-administered mice and sham-administered mice. However, the proportions of Bacteroidetes and Firmicutes differed slightly in the feces recovered at 24 hrs between *P*. *gingivalis*-administered mice and sham-administered mice, with differences being statistically significant at 48 hrs (Fig 1A). Therefore, further analysis was conducted only for fecal samples collected at 48 hrs.

OTU analysis revealed that the proportion of genus S24-7 and *Prevotella* belonging to the family Paraprevotellaceae was significantly increased in *P*. *gingivalis*-administered mice compared with sham-administered mice. In contrast, the proportion of unclassified Clostridiales belonging to the phylum Firmicutes was significantly decreased in *P*. *gingivalis*-administered mice compared with sham-administered mice (Fig 1B).

**Fig 2:**
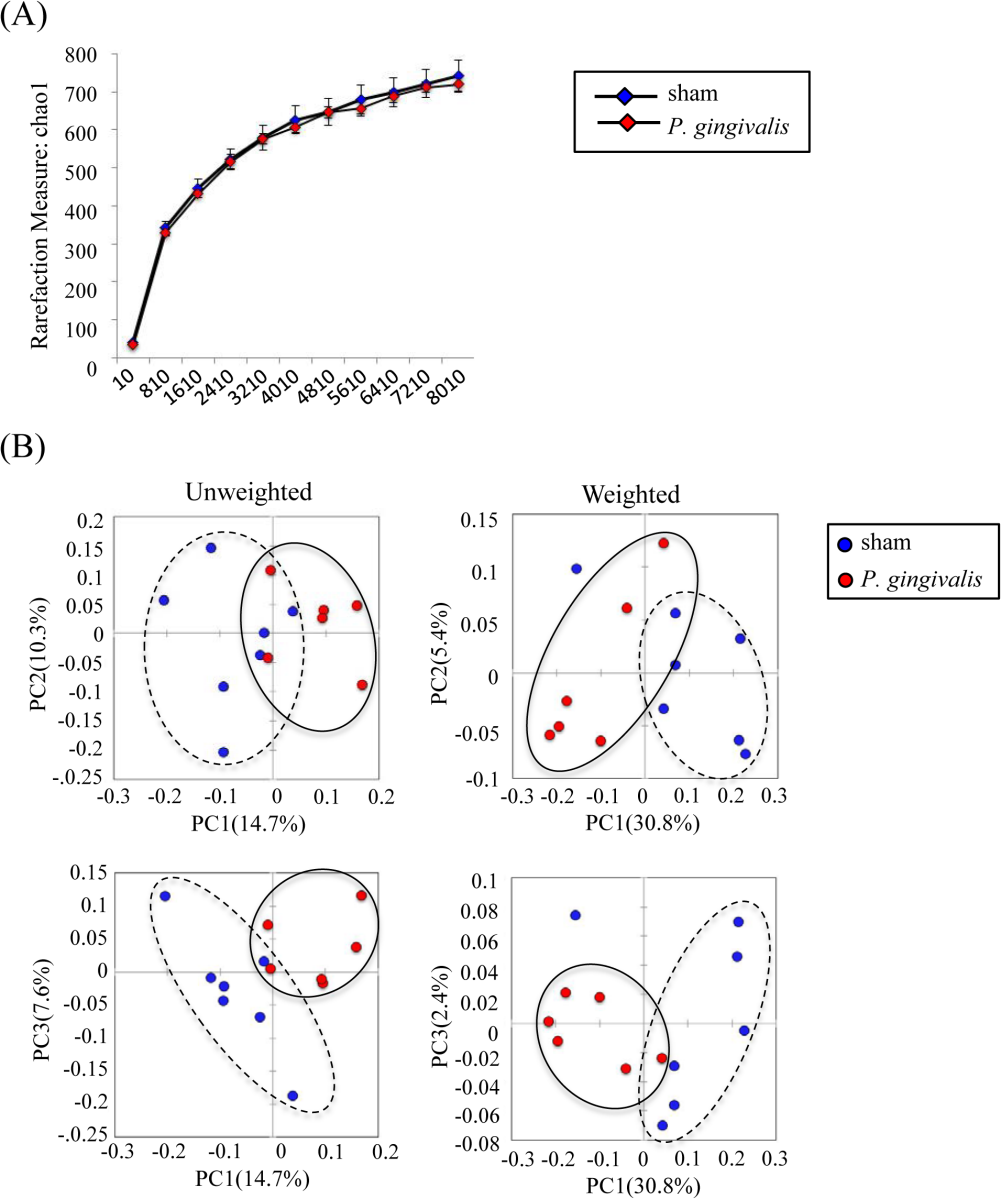
Sequencing analysis of the gut microbiota obtained from *P*. *gingivalis*-administered and sham-administered mice. (A) Diversity of bacterial species as indicated by Chao1 rarefaction measure. (B) PCoA of fecal microbiota from *P*. *gingivalis*-administered and sham-administered mice. (Unweighted distance: R = 0.2646, *p* < 0.01; Weighted distance: R = -0.0251, *p* = 0.018, ANOSIM test).

Although *P*. *gingivalis* administration did not affect the bacterial diversity in the gut (Fig 2A), principal coordinate analysis (PCoA) of unweighted and weighted UniFrac distance between samples revealed that *P*. *gingivalis* administration had a profound impact on the microbiota composition (Fig 2B).

### *P*. *gingivalis* administration decreases gut barrier function

**Fig 3:**
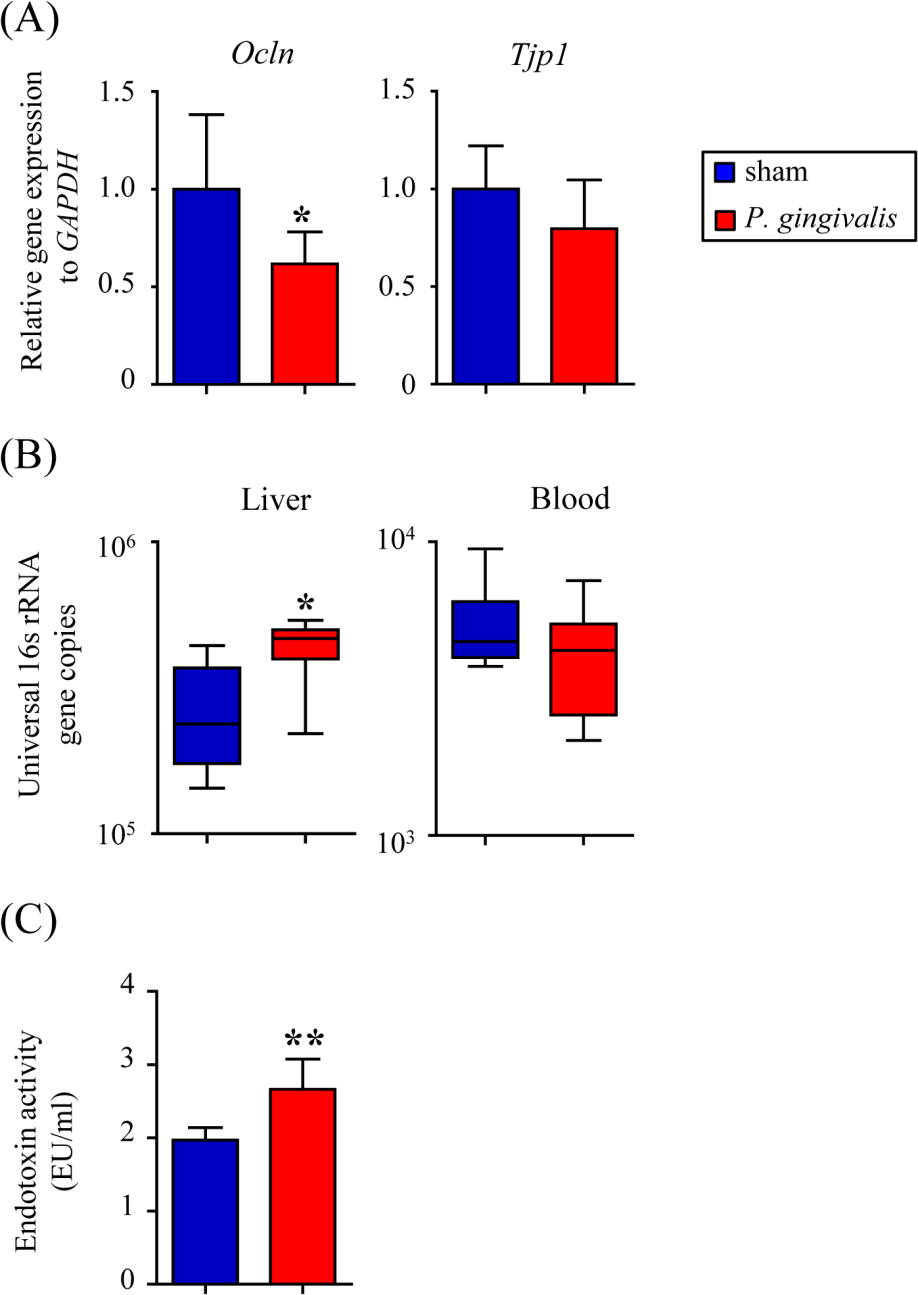
Effect of oral administration of *P*. *gingivalis* on gut barrier function and endotoxemia. (A) Quantitative PCR analysis of tight junction genes in the small intestine of *P*. *gingivalis*-administered mice and sham-administered mice at 24 hrs and 48 hrs after *P*. *gingivalis* administration. The relative mRNA expressions of the genes of interest were normalized to the relative quantity of glyceraldehyde-3-phosphate dehydrogenase (GAPDH) mRNA (N = 7 in each group). (B) Relative abundance of bacteria in the liver and blood of *P*. *gingivalis*-administered mice and sham-administered mice (N = 7 in each group). (C) Serum endotoxin (LPS) concentration (EU/mL) were determined after *P*. *gingivalis* administration or sham administration (N = 7 in each group). (*p < 0.05, **p < 0.01, Mann-Whitney U-test).

Tight junction proteins play an important role in gut barrier function. The expression of *Tjp1* and *Ocln* were downregulated in the small intestine at 48 hrs but not at 24 hrs after *P*. *gingivalis* administration, although these changes were not observed in the large intestine (Fig 3A).

In parallel with decreased mRNA expression of tight junction proteins, an influx of bacteria into the liver was evident since the copy number of the 16S rRNA gene in the liver was significantly higher in *P*. *gingivalis*-administered mice compared with sham-administered mice. In contrast, the copy number of the 16S rRNA gene in the blood did not differ between *P*. *gingivalis*-administered mice and sham-administered mice (Fig 3B). Despite this, blood endotoxin levels were significantly increased 48 hrs after *P*. *gingivalis* administration (Fig 3C). It is of note that no *P*. *gingivalis*-specific DNA was detected either in the liver or blood of *P*. *gingivalis*-administered mice (data not shown).

### *P*. *gingivalis* administration induces elevated proinflammatory gene expression in the intestine

**Fig 4:**
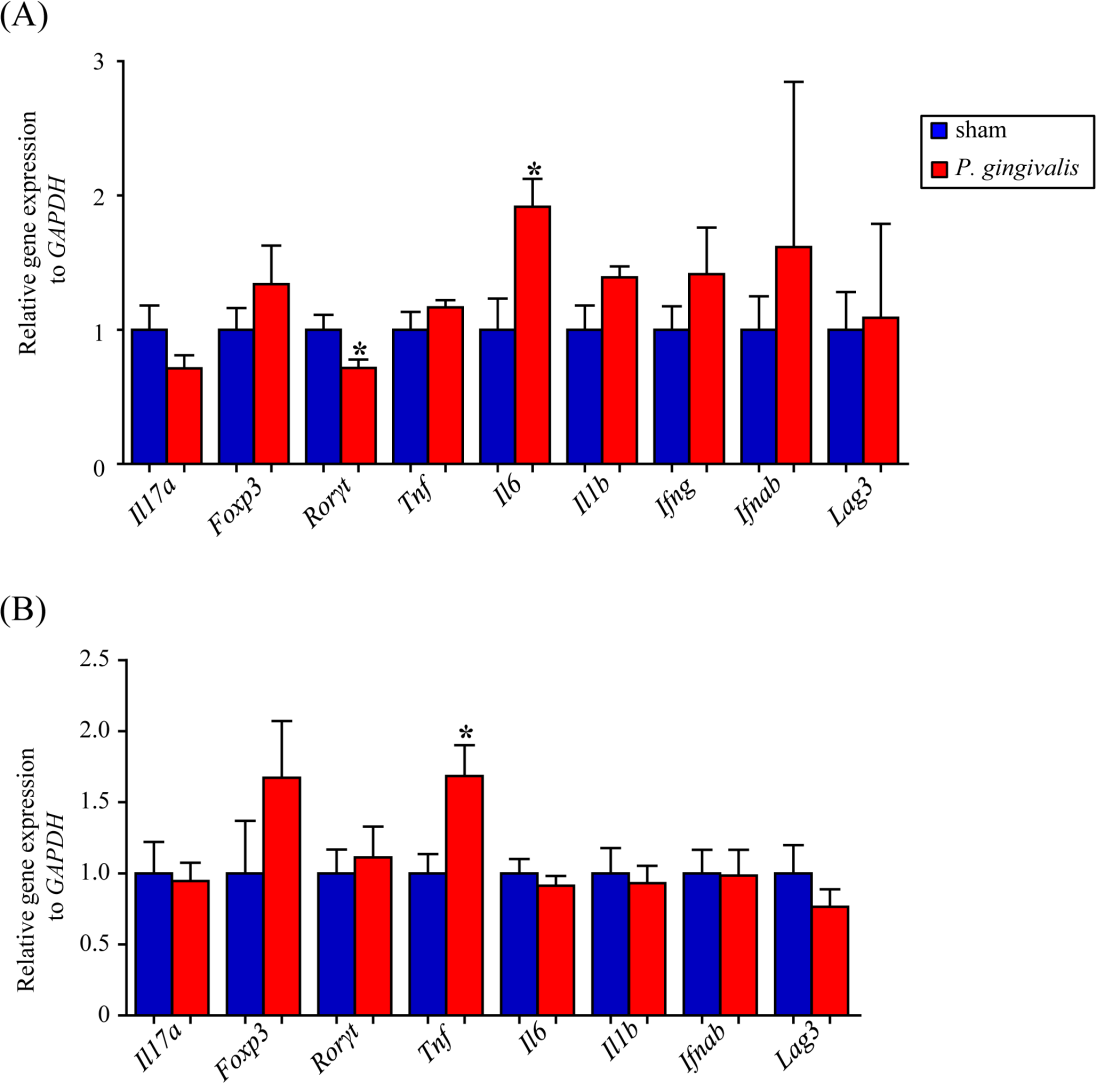
Effect of oral administration of *P*. *gingivalis* on the gene expression of the gut. (A) Relative gene expression levels in the small intestine of *P*. *gingivalis*-administered and sham-administered mice (N = 7 in each group). (B) Relative gene expression levels in the large intestine of *P*. *gingivalis*-administered and sham-administered mice (N = 7 in each group). The relative mRNA expressions of the genes of interest were normalized to the relative quantity of glyceraldehyde-3-phosphate dehydrogenase (GAPDH) mRNA. (*p < 0.05, Mann-Whitney U-test).

No change in gene expression was observed at 24 hrs after *P*. *gingivalis* administration (data not shown). At 48 hrs, IL-6 expression was significantly elevated in the small intestine of *P*. *gingivalis*-administered mice. On the other hand, mRNA expression of Rorγt, a characteristic transcription factor of Th17 cells, was significantly downregulated at this time point. Furthermore, the mRNA expressions of IFN-α and -β and of proinflammatory genes IL-1β and IFN-γ, as well as of Foxp3, a master gene of regulatory T cells, tended to be upregulated, although these differences were not statistically significant (Fig 4A).

As with the small intestine, no change in gene expression was found at 2 hrs after *P*. *gingivalis* administration (data not shown) in the large intestine. At 48 hrs, TNF-α mRNA expression was significantly increased, with a concomitant increase in Foxp3, in *P*. *gingivalis*-administered mice. However, the expression of other proinflammatory genes did not differ between the two groups (Fig 4B).

### Change in liver microbial composition after *P*. *gingivalis* administration

**Fig 5:**
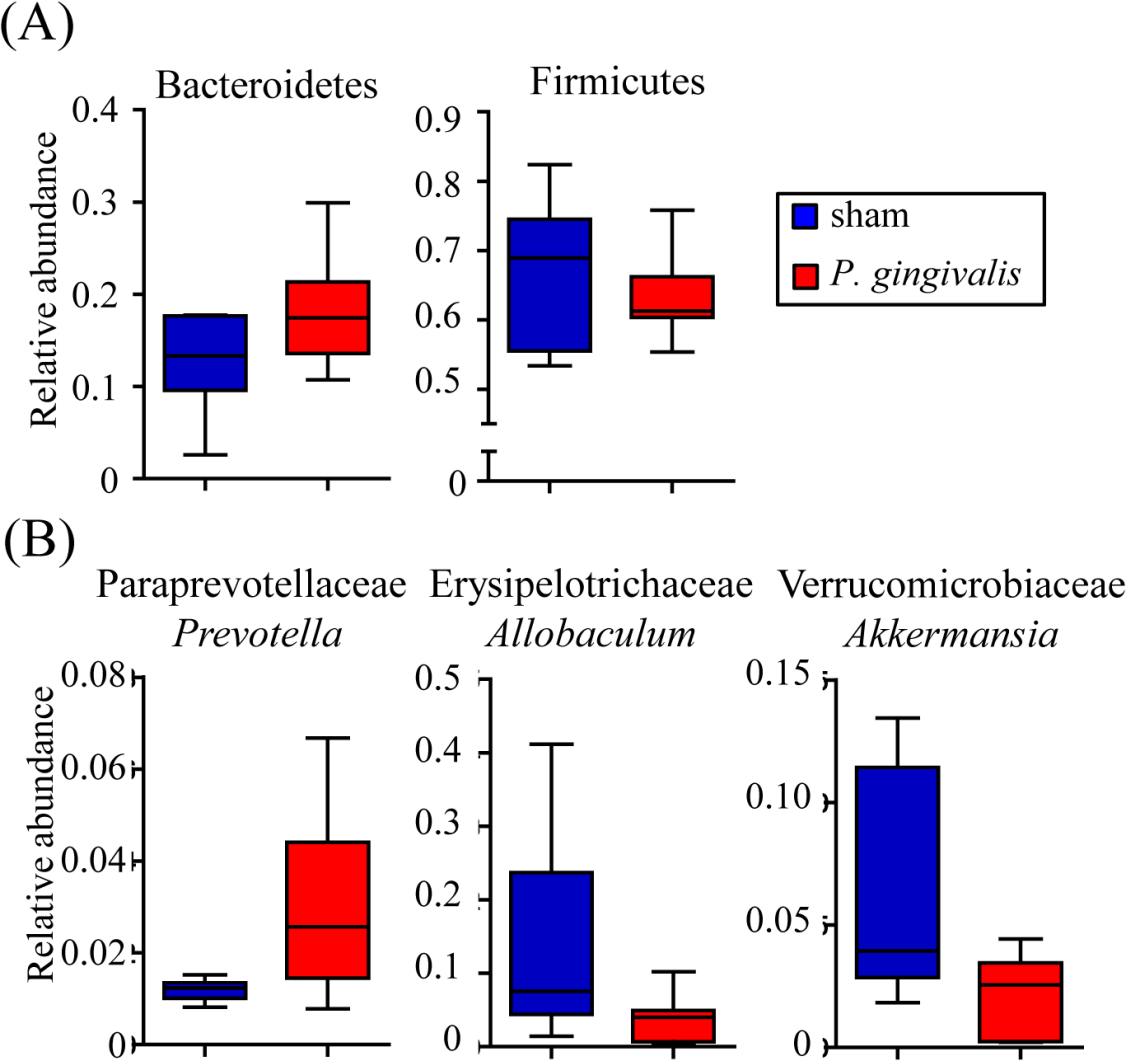
Comparison of the liver microbiota in *P*. *gingivalis*-administered and sham-administered mice by 16S rRNA sequencing analysis. (A) Relative abundances of each bacterial group at the phylum level are indicated by the box plots. (*p < 0.05, **p < 0.01, Mann-Whitney U-test). (B) Relative abundance of each bacterial group at the genus level were significantly increased or decreased are shown. Each assigned taxonomy obtained from 16S rRNA deep sequencing was compared to Silva database using UCLUST. (*p < 0.05, **p < 0.01, Mann-Whitney U-test).

**Fig 6:**
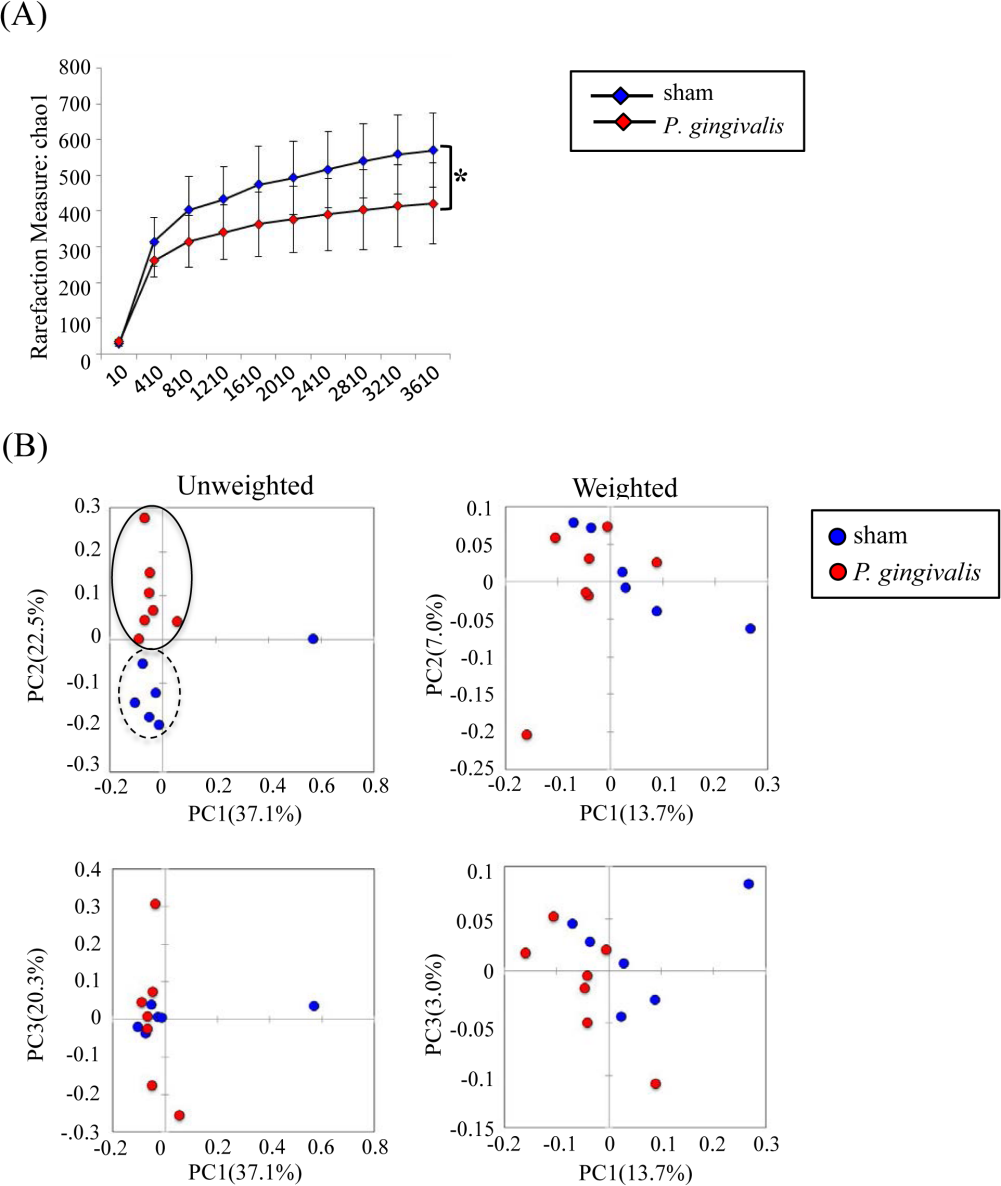
Sequencing analysis of the liver microbiota obtained from *P*. *gingivalis*-administered and sham-administered mice. (A) Diversity of bacterial species as indicated by Chao1 rarefaction measure (*p < 0.05). (B) PCoA of fecal microbiota from *P*. *gingivalis*-administered and sham-administered mice. (Unweighted distance: R = 0.3638, *p* < 0.01; Weighted distance: R = 0.4497, *p* = 0.541, ANOSIM test).

Since the copy number of the 16S rRNA gene was significantly increased after *P*. *gingivalis* administration, the composition of the microbiota was analyzed by pyrosequencing. As shown in Fig 5A, although no significant difference was found between the two groups, the phyla Bacteroidetes and Firmicutes tended to be increased and decreased, respectively, in *P*. *gingivalis*-administered mice. At the genus level, the proportion of *Prevotella* belonging to the family Paraprevotellaceae tended to be elevated, whereas those of *Allobaculum* and *Akkermansia* tended to be reduced (Fig 5B). Despite the lack of significance in these results at the individual microorganismal level, *P*. *gingivalis* administration clearly demonstrated significant impact on the liver microbiota composition and diversity (Fig 6A and 6B).

## Discussion

In this study, we found that single oral administration of *P*. *gingivalis* induced significant alteration of the phylogenetic structure and diversity of microbial communities in the gut. Moreover, this alteration of gut microbiota coincided with the dissemination of enterobacteria to the liver and decreased mRNA expression of the tight junction proteins *Tjp1* and *Ocln*, together with increased levels of serum endotoxins. Furthermore, whereas alteration of the gut microbiota composition commenced at 24 hrs, change in gene expression became evident 48 hrs after *P*. *gingivalis* administration.

Although the administered *P*. *gingivalis*-derived DNA was detected in both the fecal and cecal samples by PCR, the alteration of gut microbiota composition was not considered attributable to the administered *P*. *gingivalis* itself because the proportion of Porphyromonadaceae in the fecal samples of *P*. *gingivalis*-administered mice was less than 0.003% (data not shown). This suggests that the effect of *P*. *gingivalis* administration is quite different from other pathogenic bacteria such as *Salmonella typhimurium* that usually outgrow other indigenous bacteria. In fact, *P*. *gingivalis* has been suggested to act as a keystone pathogen for periodontal disease because *P*. *gingivalis* causes the change in the oral microbiota composition and the dramatic acceleration of alveolar bone loss despite the colonization at low level [20]. Thus, similar mechanisms seen in periodontal tissue destruction by *P*. *gingivalis* may also occur in the gut even though the precise mechanisms yet to be determined. Further studies are clearly needed to identify the molecules or metabolites derived from *P*. *gingivalis* that cause phenotypic and microbiological changes.

It has been reported that the concentration of *P*. *gingivalis* in saliva can reach 10^6^/mL in patients with severe periodontitis [21–23]. Since *P*. *gingivalis* is over-represented in the subgingival plaque of periodontitis patients[24] and its proportion in the oral flora is estimated to be 0.8% [25], and since humans produce 1–1.5 L of saliva a day, patients with severe periodontitis can be expected to swallow 10^12^–10^13^ *P*. *gingivalis* bacteria a day. Therefore, the number of bacteria administered in our mouse model seems commensurate with real conditions when the difference in body weight is taken into consideration. In addition, the administration of saliva itself from periodontitis patients and healthy subjects may provide further insight into the effect of periodontitis on systemic health.

In the fecal samples, the proportion of the phylum Bacteroidetes significantly increased, while the phylum Firmicutes significantly decreased after single administration of *P*. *gingivalis*. These changes in bacterial phyla are in accordance with those in the ileum following multiple inoculations with *P*. *gingivalis* seen in our previous study [11]. At the genus level, unclassified S24-7 and *Prevotella* belonging to the family Paraprevotellaceae were significantly elevated in *P*. *gingivalis*-administered mice. It is known that in some instances, changes in the ecological balance of the microbiota can occur such as an outgrowth of potentially pathogenic bacteria and/or a decrease in bacterial diversity, including bacteria beneficial to the host [26]. Although the pathogenicity of these bacteria has not been fully elucidated, S24-7 has been identified as being highly coated with IgA in the intestinal microbiota of mice. Palm *et al*. found that the high level of IgA coating specifically marked a select group of known inflammation- and disease-driving intestinal microbiota in mice with inflammasome-mediated colitogenic dysbiosis [27].

Similarly, it is reported that *Prevotella* species are likely involved in the transferable and colitogenic activity in a mouse dextran sulfate sodium-induced colitis model [28]. Another study demonstrated that members of the *Bacteroides/Prevotella* genuses increased together with increased inflammation in the parasite-free large intestinal tract following infection with the hookworm *Heligmosomoides polygyrus bakeri* [29]. These suggest that bacteria with increased proportion by *P*. *gingivalis* administration are associated with increased inflammation in the gut.

In contrast, the proportion of unclassified bacteria belonging to the order Clostridiales was significantly decreased in *P*. *gingivalis*-administered mice. The bacteria belonging to this taxon are associated with both health and disease [30]. Although *Clostridium difficile* is a well-known pathogenic bacterium that induces colitis [31], the identified bacteria had less than 97% similarity in the 16S rRNA gene with *Clostridium difficile* and therefore was considered to belong to a different taxon. On the other hand, it has been reported that several strains of *Clostridium* are involved in the induction of colonic regulatory T cells in mice [32]. Therefore, the reduction in the bacteria that belong to the order Clostridiales may be responsible for the proinflammatory nature of the intestine following the administration of *P*. *gingivalis*.

We also found an increased copy number of bacterial DNA in the liver of *P*. *gingivalis*-administered mice. Bacterial diversity analysis revealed that S24-7 and *Prevotella* tended to be higher in *P*. *gingivalis*-administered mice, consistent with the change in bacterial composition of the gut. Given the impaired gut barrier function, as demonstrated by the reduced mRNA expression of tight junction proteins in the small intestine, it was considered possible that altered gut microbiota could have been influxed into the liver. Although the change was not observed in the large intestine, it was also conceivable that the barrier function of the large intestine could have been impaired. In this respect, Vaziri et al. demonstrated that systemic inflammation in chronic kidney disease is associated with reductions in the protein expression of claudin-1, occluding, and ZO-1 without the change in the mRNA expression of each molecule. This suggests that post-transcriptional or post-translational modifications were a cause of the observed depletion of tight junction proteins [33]. However, no difference was found in the copy number of the 16S rRNA gene in the blood between the two groups, suggesting that reticuloendothelial cells were capable of capturing the influx of bacteria into the liver via the portal vein from the gut. The increased levels of endotoxins in the serum may also reflect impaired gut barrier function.

It has been clearly demonstrated that changes in gut microbiota can lead to metabolic endotoxemia, systemic inflammation, and associated disorders [34], likely due to an increase in intestinal permeability [35]. Increased levels of endotoxins in the serum of *P*. *gingivalis*-administered mice are not synonymous with the “metabolic endotoxemia” seen in mice fed a high-fat diet. However, repeated administration of *P*. *gingivalis* induces inflammatory changes in the adipose tissue and liver, as well as glucose intolerance, which notably are characteristics of mice on a high-fat diet [11]. Therefore, it is conceivable that oral administration of *P*. *gingivalis* has a similar effect to consuming a high-fat diet, since both induce changes in gut microbial composition and subsequent increased intestinal permeability. This notion is further supported by the similarity in the gene expression profiles of adipose tissues from high-fat diet-fed [36] and *P*. *gingivalis*-administered mice [11].

In addition to the impairment in gut barrier function, *P*. *gingivalis* administration induced increased mRNA expression of proinflammatory cytokines in the intestine. However, the reasons for the increased mRNA expression of IL-6 and TNF-α in the small intestine and large intestine, respectively, in the absence of an effect on other cytokines, are not known. Interestingly, the gene expression of Foxp3, a characteristic marker of regulatory T cells, showed a slightly but non-significant increase, whereas that of Rorγt, a characteristic marker of Th17 cells, showed a decrease. Proinflammatory cytokine genes generally showed slightly (but non-significantly) higher expression in both the small and large intestine. Although it is reasonable to suppose that the number of regulatory T cells increased to balance the increased inflammatory response in the gut tissues, the precise mechanisms for this have yet-to-be determined.

In conclusion, in the present study, we demonstrated that single oral administration of *P*. *gingivalis* induces an alteration of the gut microbiota, as well as decreased expression of tight junction protein genes in the gut. We consider these changes to be attributable to increases in endotoxins in the blood. These results provide insight into the cause-and-effect relationship of oral influx of *P*. *gingivalis* on systemic inflammation. The relationship between inflammatory changes and alteration of the gut microbiota after repeated administration of *P*. *gingivalis* may evoke the idea that the induction of inflammation in the intestine by *P*. *gingivalis* results in the alteration of the gut microbiota. However, our study clearly demonstrated that changes in gut microbiota precede any systemic inflammation as with metabolic endotoxemia by obesity-associated changes in gut microbiota [35]. Further studies will provide evidence to support this more likely hypothesis.
